# Couch potatoes do better: Delayed dispersal and territory size affect the duration of territory occupancy in a monogamous mammal

**DOI:** 10.1002/ece3.2988

**Published:** 2017-05-10

**Authors:** Martin Mayer, Andreas Zedrosser, Frank Rosell

**Affiliations:** ^1^ Faculty of Technology, Natural Sciences and Maritime SciencesDepartment of Natural Sciences and Environmental HealthUniversity College of Southeast NorwayBø i TelemarkNorway; ^2^ Department of Integrative BiologyInstitute of Wildlife Biology and Game ManagementUniversity of Natural Resources and Life SciencesViennaAustria

**Keywords:** *Castor fiber*, dispersal, Eurasian beaver, fitness, life history, territoriality

## Abstract

In territorial, socially monogamous species, the establishment and defense of a territory are an important strategy to maximize individual fitness, but the factors responsible for the duration of territory occupancy are rarely studied, especially in long‐lived mammals. A long‐term monitoring program in southeast Norway spanning over 18 years allowed us to follow the individual life histories of Eurasian beavers (*Castor fiber*) from adolescence in their natal family group to dispersal and territory establishment until the end of territory occupancy. We investigated whether territory size, resource availability, population density, and dispersal age could explain the duration of territory occupancy, which ranged from 1 to 11 years. The duration of territory occupancy was positively related to dispersal age, suggesting that individuals that delayed dispersal had a competitive advantage due to a larger body mass. This is in support with the maturation hypothesis, which states that an animal should await its physical and behavioral maturation before the acquisition of a territory. Further, we found that individuals that established in medium‐sized territories occupied them longer as compared to individuals in small or large territories. This suggests that large territories are more costly to defend due to an increased patrolling effort, and small territories might not have sufficient resources. The lifetime reproductive success ranged from zero to six kits and generally increased with an increasing duration of territory occupancy. Our findings show the importance of holding a territory and demonstrate that dispersal decisions and territory selection have important consequences for the fitness of an individual.

## 1 Introduction

**Figure 1:**
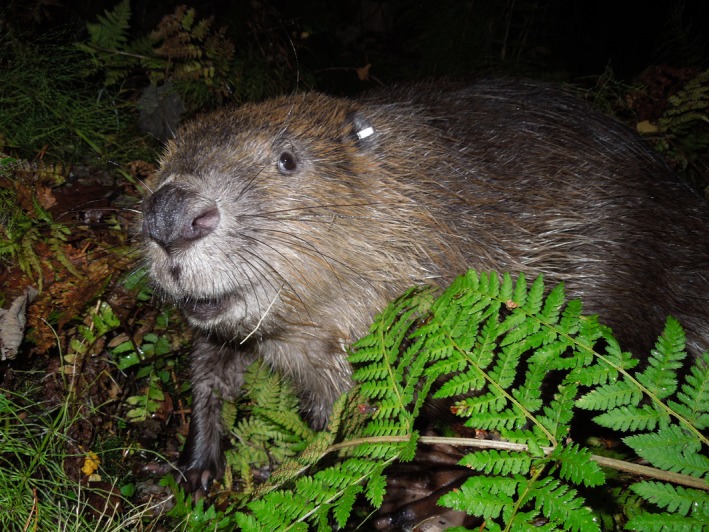
Our study species, the Eurasian beaver (*Castor fiber*) in southeast Norway

Animals have to compete for different resources, such as food, mating partners, shelter, and breeding sites, throughout their life. One way to insure access to these resources is to defend them against conspecifics, that is, being territorial (Maher & Lott, 1995). In many species with polygynous or facultative monogamous mating systems, the defense of territories is restricted to males during the reproductive season (Emlen & Oring, 1977; Hau, Wikelski, Soma, & Wingfield, 2000; Kleiman, 1977). In contrast, individuals of obligate monogamous species that rely on a partner to successfully raise offspring (Kleiman, 1977) often occupy territories year round and stay with their partners until they die or are replaced, for example, by takeover of an intruder (e.g., Lardy, Cohas, Figueroa, & Allainé, 2011; Raemaekers & Raemaekers, 1985). Here, we investigated the factors affecting the duration of territory occupancy (DTO), defined as how long (i.e., from establishment to loss of the territory) an individual is holding a territory in a large, monogamous rodent, the Eurasian beaver (*Castor fiber*, Figure 1).

The ideal despotic model predicts that the best quality individuals should monopolize the best quality territories (Fretwell & Lucas, 1970). This in turn suggests that both the quality (e.g., body size or mass) of the territory holder and the quality of the territory itself should be a predictor for the DTO (Sergio & Newton, 2003). An animal can increase its competitive ability (e.g., body mass and experience) by awaiting physical and behavioral maturity before the acquisition of a territory, that is, the maturation hypothesis (Piper et al., 2015; Weimerskirch, 1992). For example, male Siberian jays (*Perisoreus infaustus*) that delayed dispersal had more breeding events as compared to early dispersers (Ekman, Bylin, & Tegelstrîm, 1999), indicating a fitness benefit of delayed dispersal. Similarly, delayed dispersal increased the probability of survival and reproduction in red wolves (*Canis rufus*) (Sparkman, Adams, Steury, Waits, & Murray, 2010). Further, the territory size should be a predictor for the quality of the territory, and consequently the DTO, as patrolling activities are traded off with foraging activities, as shown in great tits (*Parus major*) (Ydenberg & Krebs, 1987) and chimpanzees (*Pan troglodytes*) (Amsler, 2010). Additionally, both the territory size and DTO might depend on competition with conspecifics and population density. For example, intraspecific competition influenced territory occupancy in booted eagles (*Hieraaetus pennatus*) (Martinez, Pagan, & Calvo, 2006), and in male red foxes (*Vulpes vulpes*), individuals with greater body mass held larger territories and had a higher reproductive success (Iossa, Soulsbury, Baker, & Harris, 2008) demonstrating the advantages of an increased competitive ability. Although some studies in birds investigated territory occupancy, that is, whether a territory is occupied versus unoccupied (Korpimaki, 1988; Martinez et al., 2006; Sergio & Newton, 2003), little is known about the factors affecting the DTO, especially for obligate monogamous mammals (e.g., Sparkman et al., 2010).

We used data collected from an individual‐based long‐term study of Eurasian beavers in southeast Norway to investigate the factors and mechanisms affecting the DTO. Further, we tested if DTO was a predictor for the lifetime reproductive success (LRS) of an individual, here defined as the total number of offspring produced during its lifetime. Beavers (both the Eurasian and the North American beaver [*C. canadensis*]) are large, long‐lived (up to 20 years: Gorbunova, Bozzella, and Seluanov (2008)), socially monogamous rodents that live in family groups (Jenkins & Busher, 1979; Wilsson, 1971). Both Eurasian and North American beavers typically disperse at about 2 years of age (Hartman, 1997; Sun, Müller‐Schwarze, & Schulte, 2000), but individuals were found to delay dispersal up to age seven when population densities are high (Mayer, Zedrosser, & Rosell, 2017b). Once established in an area, beavers are highly territorial (Campbell, Rosell, Nolet, & Dijkstra, 2005) and defend their territory via scent marking (Rosell, Bergan, & Parker, 1998; Rosell & Sun, 1999). Both sexes participate in territorial defense (Rosell & Nolet, 1997). Pairs remain together until the loss of a partner, either by death or emigration (Svendsen, 1989) or via replacement by a new individual (Mayer, Küenzel, Zedrosser, & Rosell, 2017a). Movement patterns of Eurasian beavers change with territory size (Herr & Rosell, 2004), and individuals trade off the costs of patrolling large territories by foraging closer to the shore (Graf, Mayer, Zedrosser, Hackländer, & Rosell, 2016). Although observed in North American beavers (Crawford, Liu, Nelson, Nielsen, & Bloomquist, 2008), there is little evidence that extrapair copulation occurs in Eurasian beavers (Syrůčková et al., 2015; Tinnesand, 2017). Thus, it is crucial for an individual's fitness, that is, its LRS, to establish and retain a territory, because only the dominant pair is reproducing in beavers (Sun, 2003).

We hypothesized that DTO in Eurasian beavers would be affected by (1) the age at dispersal and predicted that individuals that delayed dispersal would occupy a territory longer due to an increased competitive ability in comparison to younger dispersers [i.e., the maturation hypothesis: Piper et al. (2015)]. Further, we hypothesized that DTO would be related to (2) the size of the established territory, and to (3) the resource availability in the territory. We predicted that individuals in smaller territories had an increased DTO compared to individuals in larger territories due to decreased patrolling efforts (Graf et al., 2016), but only to some degree as smaller territories potentially had fewer resources (Campbell et al., 2005). We further hypothesized that (4) DTO is related to population density and predicted that individuals living at lower population densities face fewer intruders in comparison to higher densities, thus holding a territory longer. Finally, we tested the prediction that the LRS of beavers would increase with increasing DTO, while controlling for the effects of territory size, resource availability, and population density.

## 2 Methods

### 2.1 Data collection and preparation

Data were collected as part of an individual‐based long‐term study of Eurasian beavers at the rivers Straumen, Gvarv, and Saua (all emptying into Lake Norsjø) in Telemark County, southeast Norway, from 1998 to 2015. Beavers were captured annually in spring (March–June) and autumn (August–November) at night from a motor boat (Rosell & Hovde, 2001). They were individually marked with ear tags and a microchip, weighed to the closest 0.2 kg, and sex, age, and social status (dominant, subordinate [i.e., nondominant individuals ≥2 years], yearling, kit) were determined (Campbell, Nouvellet, Newman, Macdonald, & Rosell, 2012). For a detailed description of capture and handling procedures see Campbell, Newman, Macdonald, and Rosell (2013) and Rosell and Hovde (2001). Hunting pressure was considered low in all three rivers (between 1.8 and 3.3% of the population was harvested annually; FR unpublished results).

We only included individuals in this study that were captured for the first time as kit or yearling, thus, allowing exact age determination (Rosell, Zedrosser, & Parker, 2010). An individual was defined as philopatric if it remained within its natal family group for its lifetime and became dominant after the disappearance of its parents. An individual was defined as successful disperser if it left its natal territory, never returned, and established a family group outside its natal territory. We excluded individuals of unknown fate from this study. We categorized individuals that dispersed at age one to three as normal dispersers, and four years or older as delayed dispersers, based on Mayer et al. (2017b). Individuals were defined as floaters from the onset of dispersal until the establishment of a territory. Territory occupancy and dominance were determined by multiple captures and sightings in the same territory, evidence indicating the disappearance of the previous dominant individual of the same sex, lactation in females (Campbell et al., 2012), and positive paternity tests (FR, unpublished results). DTO was defined as the total number of years a dominant individual occupied a territory. The end of territory occupancy was verified either by death of the individual or via the presence of a new dominant beaver of the same sex in the territory. Apart from one exception, where a dominant beaver moved away from its original territory and occupied a new territory together with its original partner, we never observed that an individual that lost its territory established a new territory.

Territory borders were recorded based on visual observations of patrolling beavers, the presence of scent mounds (Rosell et al., 1998), and from individuals equipped with radio tags (Campbell et al., 2005) or global positioning system (GPS) (Graf et al., 2016; Steyaert, Zedrosser, & Rosell, 2015). Territory size was defined as river bank length based on territory borders determined with radio tag/GPS data in ArcMap 10.3 (Esri Redlands, CA) (Graf et al., 2016). Borders between neighboring territories were well established and barely changed over time (Campbell et al., 2005). Resource availability was calculated separately for each territory from land cover data (Felles KartDatabase, FKB data Geovekst, http://www.kartverket.no/) as the amount of mixed and deciduous forest within 50 m from the shore, following Graf et al. (2016). The annual population density was calculated as the mean number of individuals per family group separately for each river. The number of family groups per km bank length (calculated for the exact course of the bank length in ArcMap 10.3) was high in all three rivers (on average 0.64 family groups/km) with territories directly bordering each other and mostly no unoccupied areas, suggesting a saturated population (Campbell et al., 2005). The family group size and number of kits produced were recorded annually between August and October (after the kits emerged from the lodge) as part of the general population monitoring. We determined LRS by annual capture and marking of kits and positive genetic parentage analysis (FR, unpublished results). Our study might be biased toward short‐distance dispersers (<10 km) as we could not assess the DTO and reproductive success of individuals immigrating from outside our study area, because we did not know their exact age.

### 2.2 Statistical analysis

Philopatric individuals were removed from the analysis as no statistical comparison was possible due to the low sample size (*N *=* *2). Initially, we tested whether the size of the established territory (dependent variable) was related to its quality, that is, resource availability, an individual's age at dispersal (as a measure for the individual's quality) (independent variables), and the interaction of these variables using a general linear model (Table 1).

**Table 1:** Candidate models used to investigate the size of the established territory of 27 Eurasian beavers based on data collected in a population in southeast Norway between 1998 and 2015. Models were ranked based on AIC weights

Model	Variables	*df*	logLik	AIC_c_	Delta AIC	AIC weight
Size of the established territory						
1	Resource availability	24	−225.47	458.0	0.00	0.628
2	Dispersal age + Resource availability	23	−224.94	459.7	1.72	0.266
3	Dispersal age + Resource availability + Dispersal age × Resource availability	22	−224.88	462.6	4.63	0.062
4	Dispersal age	24	−228.13	463.3	5.32	0.044

To analyze which factors affect the DTO (in years, dependent variable, Poisson‐distributed), we used a generalized linear model (GLM) with a log link. As independent variables we used dispersal age, territory size (of the established territory), resource availability, and population density (averaged over the years of territory occupancy). No correlations between the independent variables were detected (all *r *<* *.6). Body mass and age at dispersal age were highly correlated (*r *=* *.76, *p *<* *.001, *N *=* *26), thus, age at dispersal was used as measure for body mass (age was used, because we did not obtain the body mass of all the individuals in the year of their dispersal, but we always knew their age) and therefore also competitive ability (Mayer et al., 2017b). Initially, we created single‐effect models for the independent variables to test if their relationship with the dependent variable was linear or quadratic, based on Akaike's Information Criterion corrected for small sample size (AIC_c_) (Hu, 2007), and found that linear function better described dispersal age, resource availability, and average population density, but that a squared function better described territory size. We then created 14 candidate models to test our hypotheses: a full model including all four independent variables without interactions, four single‐effect models for each independent variable separately, six models including the combination of two independent variables without interactions, and three models including a two‐way interaction (Table 2). The two‐way interactions were (1) territory size × dispersal age, to test whether delayed dispersers establish in smaller territories; (2) resource availability × dispersal age, to test whether delayed dispersers establish in territories with more resources; and (3) dispersal age × population density, to test if delayed dispersers had a competitive advantage when population densities were high.

**Table 2:** Candidate models for the analysis of the duration of territory occupancy of Eurasian beavers based on data collected in a population in southeast Norway between 1998 and 2015 (*N *= 19 individuals). Models were ranked based on AIC weights

Model	Variables	*df*	logLik	AICc	Delta AIC	AIC weight
Duration of territory occupancy						
1	Dispersal age + Territory size + Territory size^2^	15	−39.66	90.2	0.00	0.623
2	Territory size + Territory size^2^	16	−42.90	93.4	3.22	0.124
3	Dispersal age	17	−45.18	95.1	4.93	0.053
4	Dispersal age + Territory size + Dispersal age × Territory size	15	−42.25	95.4	5.17	0.047
5	Resource availability + Territory size + Territory size^2^	15	−42.51	95.9	5.70	0.036
6	Population density + Territory size + Territory size^2^	15	−42.82	96.5	6.31	0.027
7	Dispersal age + Population density + Resource availability + Territory size + Territory size^2^	13	−38.84	96.7	6.49	0.024
8	Dispersal age + Resource availability + Dispersal age × Resource availability	15	−43.17	97.2	7.03	0.019
9	Dispersal age + Population density	16	−44.86	97.3	7.14	0.018
10	Dispersal age + Resource availability	16	−45.11	97.8	7.65	0.014
11	Dispersal age + Population density + Dispersal age × Population density	15	−44.30	99.5	9.28	0.006
12	Resource availability	17	−47.36	99.5	9.30	0.006
13	Population density	17	−48.52	101.8	11.61	0.002
14	Population density + Resource availability	16	−47.14	101.9	11.71	0.002

We used a generalized linear mixed model (GLMM) to test whether the annual reproductive success changed with the age of the territory holder (and therefore over the time of territory occupancy). We used the number of kits produced annually as the dependent variable, the individual's age was the fixed effect (linear function fitted better), and the beaver ID was used as random effect. We used a negative binomial response distribution in the R package *glmmADMB* (Bolker, Skaug, Magnusson, & Nielsen, 2012). To analyze LRS (dependent variable, Poisson‐distributed), we used a GLM with a log link. Independent variables were the quadratic function of DTO, resource availability, mean population density, and the quadratic function of territory size; the variables were not correlated with each other (all *r *<* *.6). We then created a set of 11 candidate models (Table 3).

**Table 3:** Candidate models used for the analysis of the lifetime reproductive success of Eurasian beaver in a population in southeast Norway between 1998 and 2015 (*N *= 25 individuals). Models were ranked based on AIC weights

Model	Variables	*df*	logLik	AICc	Delta AIC	AIC weight
Lifetime reproductive success						
1	DTO + DTO^2^	3	−44.31	95.8	0.00	0.466
2	DTO + DTO^2^ + Resource availability	4	−43.44	96.9	1.12	0.267
3	Population density + DTO + DTO^2^	4	−43.72	97.4	1.68	0.201
4	DTO + DTO^2^ + Territory size + Territory size^2^	5	−43.35	99.9	4.09	0.060
5	DTO + DTO^2^ + Population density + Resource availability + Territory size + Territory size^2^	7	−42.18	104.9	9.19	0.005
6	Territory size + Territory size^2^	3	−52.36	111.9	16.11	0.000
7	Resource availability	2	−53.77	112.1	16.32	0.000
8	Population density	2	−53.77	112.1	16.33	0.000
9	Resource availability + Territory size + Territory size^2^	4	−52.26	114.5	18.75	0.000
10	Population density + Resource availability	3	−53.76	114.7	18.91	0.000
11	Population density + Territory size + Territory size^2^	4	−52.35	114.7	18.94	0.000

DTO, duration of territory occupancy.

Model selection of all analyses was based on AIC_c_ and Akaike weights (Wagenmakers & Farrell, 2004), and parameter estimates that included zero within their 95% confidence interval (CI) were considered as uninformative (Arnold, 2010). If ∆AIC_c_ was <4 in two or more of the most parsimonious models, we performed model averaging (Anderson, 2008). Sample sizes in the different analyses vary because we did not always have complete information for all individuals (Table S1). All statistical analyses were performed in R 3.2.1 (R Core Team, 2015).

## 3 Results

**Figure 2:**
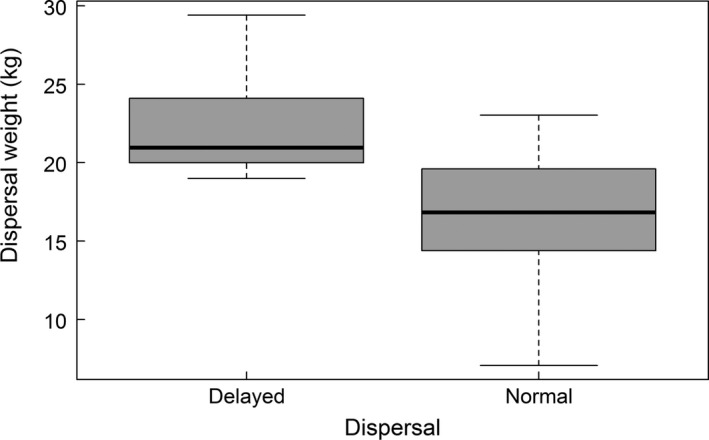
Boxplot of the body mass of Eurasian beavers in the year of dispersal for delayed (≥4 years old, *N *= 14) and normal (1–3 year old, *N *= 20) dispersers in southeast Norway. The boxplot shows median values, and 25th and 75th percentile and 95% confidence intervals

Annual population density varied between 3.1 and 5.1 individuals per family group (mean ± SD = 3.7 ± 0.6) and territory sizes varied between 1.4 and 5.6 km bank length (3.6 ± 1.2 km). Normal dispersers (*N *=* *23) dispersed at a mean age of 2.35 ± 0.71 years old and delayed dispersers at a mean age of 5.06 ± 0.93 years (*N *=* *16); the average age at dispersal for both groups combined was 3.5 ± 1.6 years. Delayed dispersers (*N *=* *14) had a significantly greater body mass than normal dispersers (*N *=* *20) in the year of dispersal (22.1 ± 3.0 vs. 16.8 ± 4.5 kg, *t* test: *p *<* *.001, Figure 2). The size of the established territory was positively related to the amount of mixed and deciduous forest (*N *=* *27, Tables 1 and 4).

**Table 4:** Effect size (β), standard error (SE), lower (LCI) and upper (UCI) 95% confidence interval of explanatory variables for the analyses of the size of the established territory, the duration of territory occupancy, and the lifetime reproductive success in a Eurasian beaver population in southeast Norway between 1998 and 2015. We performed model averaging of best models (∆AIC_c_ < 4) to estimate the effect size of each variable. Informative parameters are given in bold

Parameter	Estimate	*SE*	LCI	UCI
Size of the established territory				
**Resource availability**	**0.015**	**0.005**	**0.005**	**0.026**
Dispersal age	−141.500	144.700	−440.273	157.184
Duration of territory occupancy				
**Dispersal age**	**0.147**	**0.057**	**0.026**	**0.268**
Territory size	**1.827**	**0.713**	**0.315**	**3.339**
**Territory size^2^**	−**0.275**	**0.099**	−**0.486**	−**0.065**
Lifetime reproductive success				
	**1.013**	**0.399**	**0.185**	**1.841**
**Duration of territory occupancy^2^**	−**0.060**	**0.027**	−**0.117**	−**0.003**
Population density	−0.234	0.220	−0.692	0.224
Resource availability	0.000	0.000	0.000	0.000

### 3.1 Duration of territory occupancy

**Figure 3:**
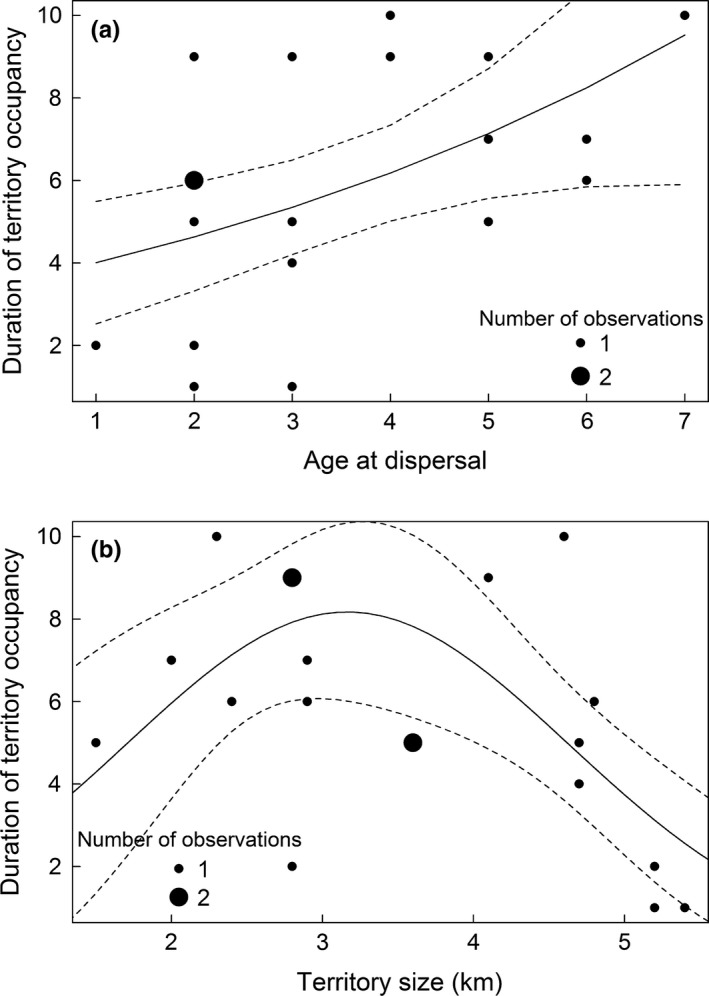
The predicted relationship (solid line) between (a) the age at dispersal (in years) and the duration of territory occupancy (DTO, in years), and (b) the territory size and DTO for 19 Eurasian beavers in southeast Norway (1998–2015). Dashed lines present the upper and lower 95% confidence interval

We had data of 25 individuals from 16 different territories with known and finished DTO (Table S1). Two individuals remained philopatric and became dominant in their natal territory after their parents had disappeared; the remaining 23 beavers dispersed and established in a new territory. Of the dispersers, 11 individuals were normal dispersers (1–3 years old) and eight were delayed dispersers (≥4 years old) (the dispersal age of the remaining four beavers was unknown). DTO ranged from one to eleven years, and mean DTO was not significantly different between females (6.3 ± 2.5 years, *N *=* *12) and males (6.1 ± 3.2 years, *N *=* *13, *t* test: *p *=* *.948). Two individuals (8%) were killed by a vehicle, seven (28%) were killed by hunters, and the cause of disappearance in the remaining 16 beavers was unknown (64%). There was no significant difference in DTO between individuals that died due to human‐caused mortalities and ones with unknown causes of disappearance (5.7 ± 2.7 vs. 6.5 ± 2.9 years, *t* test: *p *=* *.485). DTO was best explained by the territory size and age at dispersal (Tables 2 and 4): Beavers that delayed dispersal and established in medium‐sized territories occupied their territory longer than normal dispersers and individuals in smaller or larger territories (Figure 3).

### 3.2 Annual reproduction and lifetime reproductive success

**Figure 4:**
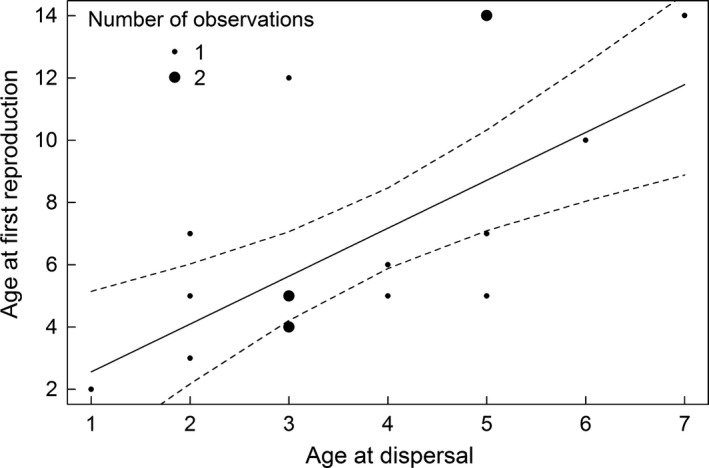
The predicted relationship (solid line) between the age at dispersal (in years) and the age at first reproduction (in years) for 18 Eurasian beavers in southeast Norway (1998–2015). Dashed lines present the upper and lower 95% confidence interval

**Figure 5:**
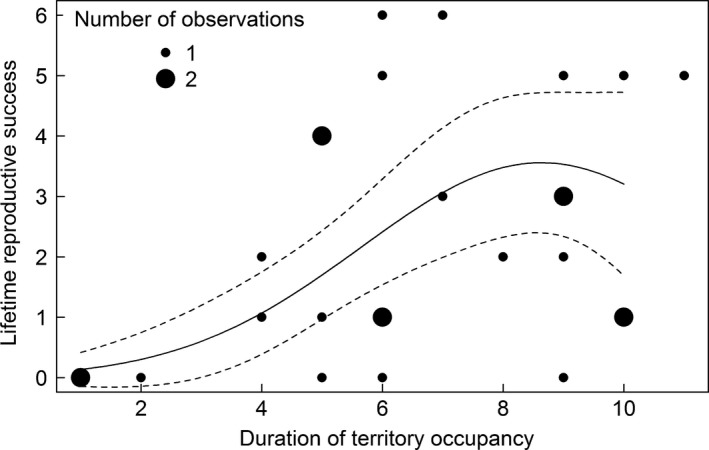
The predicted relationship (solid line) between the duration of territory occupancy (in years) and the lifetime reproductive success (measured as total number of kits produced in a lifetime) for 25 Eurasian beavers in southeast Norway (1998–2015). Dashed lines present the upper and lower 95% confidence interval

Of 35 dominant individuals (of which 10 were still alive when we drafted this manuscript), 25 produced offspring. Individuals that dispersed at older ages were also older when reproducing for the first time (β = 1.537 ± 0.404, 95% CI: 0.745; 2.329, *N *=* *18, Figure 4). The annual reproductive success ranged from zero to four kits (0.63 ± 0.97, median = 0) and decreased with the age of the territory holder (β = −0.10 ± 0.04, 95% CI: −0.18; −0.02, *N *=* *25, 163 individual years) with older individuals producing fewer kits. The LRS was known for 25 individuals and ranged from zero to six kits (2.2 ± 2.1, median = 2). LRS was best explained by the squared function of DTO (Tables 3 and 4). The LRS increased with increasing DTO, but then leveled off in individuals that held a territory for more than 9 years (Figure 5).

## 4 Discussion

We investigated the factors best explaining the duration of territory occupancy (DTO) and lifetime reproductive success (LRS) in the Eurasian beaver and found that there was a positive relationship between these two measures, with individuals holding a territory longer also having a higher LRS. This is in line with Sergio and Newton (2003) who suggest that territory occupancy is a measure for territory quality and thus also individual fitness. DTO was longer for individuals dispersing at an older age, indicating that delayed dispersal may be a strategy to increase individual fitness, similar to findings in red wolves (Sparkman et al., 2010) and Siberian jays (Ekman et al., 1999). Further, the DTO was affected by the size of the established territory, indicating a trade‐off between the costs of patrolling larger territories and possibly limited resource availability in smaller territories. This is in line with the optimization criterion that predicts a time constraint between foraging and territory defense (Adams, 2001). Consequently, this trade‐off should result in a minimum economically defensible area (Adams, 2001).

### 4.1 The duration of territory occupancy

#### 4.1.1 Dispersal age

In populations below carrying capacity, dispersal at a young age is presumably the best strategy as competition is low, and the chances to establish an own territory are high. For example, prairie voles (*Microtus ochrogaster*) dispersed at younger ages when population densities were lower (McGuire, Getz, Hofmann, Pizzuto, & Frase, 1993). In contrast, high‐density populations likely exert a strong selection on the competitive ability (e.g., body condition and perhaps experience) (Mueller, 1988) of dispersers that try to establish and defend a territory, and Ekman et al. (1999) suggested that there should be a competitive advantage for individuals that delay dispersal.

In our saturated population (Campbell et al., 2005), 41% of the beavers delayed dispersal, and individuals that were older at dispersal had a greater DTO in comparison to younger dispersers. Beavers that spent a longer period within their natal family group had a greater body mass at the time of dispersal, and delayed dispersers were on average 31% heavier than normal dispersers. This likely resulted in a competitive advantage to establish and retain a territory. Generally, beavers in our study area do not reach their maximum body mass before age six (Mayer et al., 2017a). Hence, individuals that established at younger ages were probably more prone to lose their territory to larger individuals before reaching their maximum body mass. In the three‐spined stickleback (*Gasterosteus aculeatus*), larger males were more successful in obtaining and defending a territory (Rowland, 1989), and in Common loons (*Gavia immer*), larger individuals held mating territories longer, possibly due to an increased fighting ability (Piper, Tischler, & Klich, 2000). Additionally, by remaining longer in the natal family group, an individual might gain parenting experience, for example, via helper behavior (Cockburn, 1998) as subordinate North American beavers were shown to provision kits with food before they emerge from the lodge (Müller‐Schwarze & Sun, 2003). Further, subordinates might gain experience in patrolling and defending the territory (subordinates were occasionally shown to overmark scent mounds [Tinnesand, Jojola, Zedrosser, & Rosell, 2013; Wilsson, 1971)], and they might gain experience in lodge building and food caching. Our findings are in support of the maturation hypothesis which states that an animal should await physical and behavioral maturity before the acquisition of a territory (Piper et al., 2015; Weimerskirch, 1992).

If there is a fitness benefit for delayed dispersers, the question arises why not all individuals delay dispersal. Parental tolerance toward the offspring is assumed to be a driver for the evolution of delayed dispersal (Ekman, Sklepkovych, & Tegelstrom, 1994), and the age at dispersal in our study area was positively related to the parental age (Mayer et al., 2017b). Older parents might be more tolerant toward their offspring (Graf et al., 2016; Mayer et al., 2017b), whereas younger parents might force their offspring to disperse earlier. Alternatively, individuals might perceive senescence in their parents and await their disappearance in order to take over the natal territory as shown in female common lizards (*Lacerta vivipara*) (Ronce, Clobert, & Massot, 1998).

#### 4.1.2 Territory size

Apart from the dispersal age, DTO was related to territory size, with individuals establishing in medium‐sized territories having a greater DTO as individuals in smaller and larger territories. This indicates that medium‐sized territories were of better quality, either via a higher resource availability (compared to smaller territories) or a decreased patrolling effort (compared to larger territories), or both. In a beaver population in southern France, smaller territories had a higher willow (*Salix* sp.) grove cover as compared to larger ones (however, these results must be treated with caution, because there were substantial limitations to the estimation of territory sizes) (Fustec, Lodé, le Jacques, & Cormier, 2001). However, two other studies (of which one was conducted in our study area) found that larger territories had a higher percentage of deciduous habitat, implying that larger territories were of better habitat quality (Campbell et al., 2005; McClintic, Taylor, Jones, Singleton, & Wang, 2014). The important question is which parameters are in fact decisive for the quality of a territory? The above‐mentioned studies (and the current study) only used mixed and deciduous woody plants as measure for quality. However, especially from late spring to early fall, grass, herbs, and aquatic plants can play an important dietary role, as shown in North American beavers (Milligan & Humphries, 2010), and Fryxell (2001) showed that beaver density was positively related to aquatic biomass. Unfortunately, we did not have a reliable measure for nonwoody plants, especially over the long time scale of the study, and resource availability could change over time, for example, due to resource depletion (Beier & Barrett, 1987; Fryxell, 2001). Due to these difficulties, the measure of territory size might provide a possibility to potentially bypass arduous habitat studies (Sergio & Newton, 2003). We did not have evidence that delayed dispersers, which are larger and heavier than normal dispersers, established in smaller territories. The reason could be, because in saturated populations, territories that become vacant, will be re‐occupied independently of their size (Campbell et al., 2005).

Apart from being an estimate for resource availability, the territory size can entail a trade‐off between foraging and territorial defense. For example, in red‐backed salamanders (*Plethodon cinereus*), territorial defense constrained the time spent foraging and the quality of food items consumed, independent of resource availability (Jaeger, Nishikawa, & Barnard, 1983), and male chimpanzees spent more time traveling and less time foraging when on patrolling trips (Amsler, 2010). However, these studies did not provide information on long‐term or fitness effects of territorial defense. In our study area, beavers were foraging closer to the shore when occupying larger territories as compared to smaller ones, suggesting sufficient resource availability in larger territories (Graf et al., 2016). It was shown that beavers in larger territories were moving greater distances in water and spent more time patrolling, indicating that they used more energy due to swimming effort and temperature constrains (being in cold water) (Graf et al., 2016; Herr & Rosell, 2004). Hence, there is evidence that the defense of larger territories is constrained by an increased patrolling effort, which in effect leads to a reduced DTO and LRS. In line with our findings, a study in sanderlings (*Calidris alba*) found that territories with better resource (prey) availability had increased intruder frequencies, consequently leading to smaller territory sizes due to the high costs of territorial defense (Myers, Connors, & Pitelka, 1979). After controlling for the interaction of prey density and intruder pressure, prey density had no effect on the territory size, emphasizing the importance of territorial defense for the optimal territory size (Myers et al., 1979). In conclusion, it appears that the intermediate territory sizes follow the optimization criterion stating that there is a time constraint between foraging and territory defense resulting in a minimum economically defensible area (Adams, 2001; Gill & Wolf, 1975).

#### 4.1.3 The end of territory occupancy

Although having a low effect at the population level (between 2% and 3% annually), hunting mortality might play an important role for individual dominant territory holders, as 28% of all mortalities in this study were caused by hunting, and because there appears to be a selection for adults and pregnant females (Parker, Rosell, Hermansen, Sørløkk, & Stærk, 2002). However, there was no difference in DTO between individuals that died due to human causes and individuals of unknown disappearance, but these results must be treated with caution due to our small sample size. We have evidence that individuals of unknown fate were forced out by an intruding individual of the same sex that took over the territory (Mayer et al., 2017a). Alternatively, human‐caused mortality might be more common as observed, due to unreported cases of hunting or poaching, for example, shown in wolves (*Canis lupus*) (Liberg et al., 2012; Milleret et al., 2016). After the loss of its territory, an individual could have died, it could have become a floater, or it could have managed to establish in a new territory with a new mate. The first two possibilities would not increase the individuals’ fitness, whereas secondary dispersal and territory occupancy could increase the total DTO and LRS. Although secondary dispersal was found in 39% of all dispersal events in a study of North American beavers (Sun et al., 2000), we observed secondary dispersal in only one case (both members of a pair moved to a new territory). Generally, secondary dispersal seems very unlikely in our study area due to the saturated population with few unoccupied territories available (Campbell et al., 2005). Nevertheless, we cannot exclude that beavers were establishing outside our study area.

### 4.2 Lifetime reproductive success

Dispersal at an older age may be costly in terms of lifetime reproductive success as it represents a trade‐off with an increased age at first reproduction. Additionally, the reproductive success decreased with increasing age of the territory holder, indicating senescence. However, these costs were offset by an increased DTO in delayed dispersers, resulting in a greater LRS. Apart from increasing their body mass and thus, competitive ability, delayed dispersers might additionally gain parenting skills that later increase their reproductive success (Cockburn, 1998). Our finding that senescing beavers produce fewer offspring could explain why the LRS leveled off after a certain time of territory occupancy. Further, territories that had been occupied for a longer time might have suffered from a greater resource depletion, which could result in a lower reproductive success. Finally, there is natural variation in the reproductive success between individuals (Kruuk, Clutton‐Brock, Rose, & Guinness, 1999; Pelletier, Clutton‐Brock, Pemberton, Tuljapurkar, & Coulson, 2007), suggesting that some beavers may have contributed more to population growth despite having a shorter DTO. Similar to our study, it was shown that delayed dispersal could lead to an increased LRS in male Siberian jays (Ekman et al., 1999) and to an increased probability of reproduction in male red wolves (Sparkman et al., 2010). Generally, the annual reproductive success in our study area was very low compared to other Eurasian beaver populations (Halley, 2011; Saveljev & Milishnikov, 2002). This might be due to resource depletion as beavers inhabited the area at least since 1920 (Olstad, 1937) and because the population is saturated (Campbell et al., 2012; Steyaert et al., 2015). Alternatively, the low reproductive success might be caused by an inbreeding depression, because the genetic diversity in our population is lower as compared to other populations (Durka et al., 2005).

## 5 Conclusion

Studies investigating factors affecting DTO in long‐lived mammals are rare (e.g., Sparkman et al., 2010), possibly due to the challenge of compiling detailed long‐term individual‐based data sets. Nevertheless, such data are necessary to answer important questions in ecology and evolution (Clutton‐Brock & Sheldon, 2010). Here, we show evidence that delayed dispersal and the establishment in intermediate sized territories provided fitness benefits in beavers, such as increased DTO and LRS. Intermediate territories follow the optimization criterion (Adams, 2001), insuring sufficient resource availability and decreased costs of territorial defense at the same time. Further, we could demonstrate a competitive benefit of delayed dispersal due to increased body mass, as suggested by Ekman et al. (1999).

## Conflict of Interest

None declared.

## Author Contributions

MM, AZ and FR developed the design of the work, MM and FR contributed to the data collection, MM and AZ performed the statistical analyses, and MM wrote the manuscript.
