# Supplementary material for: Couch potatoes do better: Delayed dispersal and territory size affect the duration of territory occupancy in a monogamous mammal
Source: Ecol Evol. 2017 May 10;7(12):4347–56. doi: 10.1002/ece3.2988 (PMC5478066; doi:10.1002/ece3.2988)
Supplement: Supplementary file 1 [file ECE3-7-4347-s001.docx]

**Supplementary material**

Table S1: Overview of 26 Eurasian beavers with complete life history in a population in southeast Norway between 1998 and 2015.

| Name | Sex | DTO* | LRS** | Age at dispersal | Status | Age at fist reproduction | Average population density (individuals per colony) | Body mass at dispersal (kg) | Average colony size | Territory size (km) |
| --- | --- | --- | --- | --- | --- | --- | --- | --- | --- | --- |
| Alfhild | F | 1 | 0 | 2 | Normal disperser |  | 3.5 | 14.9 | 2.0 | 5.4 |
| Andreas | M | 1 | 0 | 3 | Normal disperser |  | 3.6 | 19.8 | 2.0 | 5.2 |
| Anne Lise | F | 9 | 5 | 2 | Normal disperser | 5 | 3.7 | 17.7 | 3.3 | 4.1 |
| Bram | M | 10 | 1 | 7 | Delayed disperser | 14 | 3.3 | 24.1 | 3.2 | 4.6 |
| Chris | M | 4 | 1 |  | Philopatric | 11 | 3.2 |  | 3.5 | 2.4 |
| Demi | F | 9 | 2 | 3 | Normal disperser | 12 | 4 | 22.7 | 2.3 | 2.8 |
| Easy | M | 7 | 3 | 6 | Delayed disperser | 10 | 3.2 | 21.1 | 3.0 | 2.0 |
| Greg burly | M | 9 | 0 | 4 | Delayed disperser |  | 4 | 22.8 | 2.3 | 2.8 |
| Hanna Christi | F | 5 | 0 | 2 | Normal disperser |  | 3.2 | 12.8 | 2.0 | 3.6 |
| Hanne | F | 7 | 3 |  | Philopatric | 6 | 3.8 |  | 3.7 | 4.3 |
| Homer | M | 6 | 1 | NA | Unknown | 5 | 4.6 | NA | 3.2 | 1.5 |
| Ida | F | 4 | 2 | 3 | Normal disperser | 5 | 3.6 | 19 | 4.3 | 4.7 |
| Karin | F | 6 | 6 | 2 | Normal disperser | 3 | 3.5 | 14.2 | 3.8 | 4.8 |
| Kathrin | F | 8 | 2 | NA | Unknown | 5 | 5.2 | NA | 2.8 | 1.5 |
| Kolbjørn | M | 2 | 0 | 2 | Normal disperser |  | 3.5 | 16.5 | 2.0 | 5.2 |
| Konrad | M | 6 | 5 | 2 | Normal disperser | 7 | 5.1 | 15 | 5.0 | 2.9 |
| Linn | F | 6 | 0 | NA | Unknown |  | 4.9 | NA | 2.3 | 2.8 |
| Loran | M | 7 | 6 | 5 | Delayed disperser | 7 | 3.2 | 24.3 | 3.1 | 2.9 |
| Maerta | F | 6 | 1 | 6 | Delayed disperser | 10 | 3.2 | 26.1 | 4.0 | 2.4 |
| Montana | M | 2 | 0 | 1 | Normal disperser |  | 3.1 | 8.7 | 2.0 | 2.8 |
| Oddi | M | 5 | 1 | 5 | Delayed disperser | 14 | 5.1 | NA | 2.2 | 1.5 |
| Ola By | M | 11 | 5 | NA | Unknown | 7 | 4 | NA | 4.3 | 1.9 |
| Stina | F | 10 | 5 | 4 | Delayed disperser | 5 | 3.6 | 20.2 | 4.7 | 2.3 |
| Suzanne | F | 5 | 4 | 3 | Normal disperser | 5 | 3.3 | 18.2 | 3.4 | 4.7 |
| Terje | M | 9 | 3 | 5 | Delayed disperser | 5 | 3.7 | 19.6 | 3.3 | 4.1 |
| Darwin | M | NA | NA | 2 | Normal disperser |  | 3.1 | 14.8 | 2.0 | 4.8 |
| Jodie | F | NA | NA |  | Philopatric | 8 | 5 |  | 5.2 | 4.3 |
| Lasse | M | NA | NA | 1 | Normal disperser | 2 | 3.3 | 7.1 | 4.8 | 5.4 |
| Laurits | M | NA | NA | 4 | Delayed disperser | 6 | 4.1 | 19 | 5.8 | 4.8 |
| Leigh | F | NA | NA | 5 | Delayed disperser |  | 4.1 | 29.4 | 2.6 | 1.8 |
| Live | F | NA | NA |  | Philopatric | 4 | 4.3 |  | 5.0 | 2.3 |
| Lona | F | NA | NA | 4 | Delayed disperser | 5 | 3.9 | 20 | 3.1 | 4.3 |
| Morten | M | NA | NA | 3 | Normal disperser | 4 | 4.3 | 19.8 | 5.0 | 5.7 |
| Paddy | M | NA | NA | 3 | Normal disperser | 4 | 4.7 | 23 | 6.3 | 2.4 |
| Sara | F | NA | NA | 2 | Normal disperser |  | 3.4 | 23 | 2.0 | 2.4 |

* DTO = duration of territory occupancy

** LRS = lifetime reproductive success
